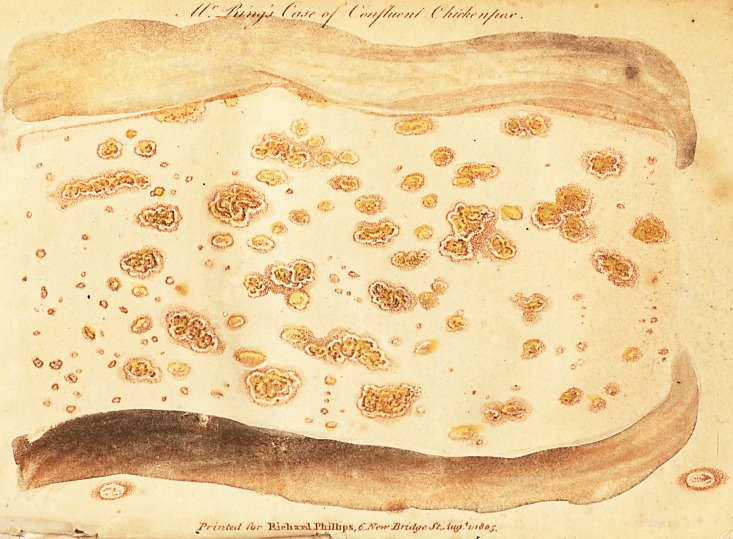# Mr. Ring's Case of Confluent Chicken-Pox

**Published:** 1805-08-01

**Authors:** John Ring

**Affiliations:** New Street, Hanover Square


### *To the Editors of the Medical and Physical Journal.*

GENTLEMEN,

THE following is an explanation of the drawing, which I some time ago transmitted to you, representing a case of confluent chicken-pox. It occurred in a boy about four years of age, the son of Mr. Warren, at No. 6, Husband Street, Carnaby Market. It was seen by Dr. Denman and Mr. Newby; both of whom declared, that they had never before known a case of chicken-pox attended with so copious an eruption. The drawing was made by that celebrated artist Mr. Edwards, from the back of the child, about the sixth day of the eruption; and a few vesicles which appear below the large drawing, were taken from another child in the same family, who had the same disorder in the usual manner, at the same time; partly for the sake of shewing the more common appearance of the chicken-pox, and partly for the sake of proving that the confluent case really was that disease. Dr. Willan has a drawing of a case of chicken-pox, in which the eruption is still more numerous, but smaller and less confluent.

Mr. Warren's son had been vaccinated some time before fore; and when the chicken-pox broke out in so formidable a manner, it was mistaken for the small-pox. On this account, I was informed of the case. His mother told me, that when this eruption first appeared, he was scarcely recovered from the measles; and she was of opinion, that the chicken-pocks succeeded in every place where the measles had before appeared.

*New Street, Hanover Square,*

*July* 16, 1805.

I am, &c.
JOHN RING.

Mr. Ring's Case of Confluent Chickenpox.

## Figures and Tables

**Figure f1:**